# Male mice with deleted Wolframin (Wfs1) gene have reduced fertility

**DOI:** 10.1186/1477-7827-7-82

**Published:** 2009-08-10

**Authors:** Klari Noormets, Sulev Kõks, Ants Kavak, Andres Arend, Marina Aunapuu, Aivi Keldrimaa, Eero Vasar, Vallo Tillmann

**Affiliations:** 1 Department of Paediatrics, University of Tartu, 6 Lunini Street, 51014 Tartu, Estonia; 2 Department of Physiology, University of Tartu, 19 Ravila Street, 50411 Tartu, Estonia; 3 Institute of Veterinary Medicine and Animal Sciences, Estonian University of Life Sciences, 62 Kreutzwaldi Street, 51014 Tartu, Estonia; 4 Department of Anatomy, Chair of Histology and Embryology, University of Tartu, 19 Ravila Street, 50411 Tartu, Estonia

## Abstract

**Background:**

Wolfram Syndrome (WS) is an autosomal recessive disorder characterised by non-autoimmune diabetes mellitus, optic atrophy, cranial diabetes insipidus and sensorineural deafness. Some reports have described hypogonadism in male WS patients. The aim of our study was to find out whether Wfs1 deficient (Wfs1KO) male mice have reduced fertility and, if so, to examine possible causes.

**Methods:**

Wfs1KO mice were generated by homologous recombination. Both Wfs1KO and wild type (wt) male mice were mated with wt female mice. The number of litters and the number of pups were counted and pregnancy rates calculated. The motility and morphology of the sperm and the histology of testes were analysed. Serum testosterone and FSH concentrations were also measured.

**Results:**

The pregnancy rate in wt females mated with Wfs1KO males was significantly lower than in the control group (15% vs. 32%; p < 0.05), but there was no significant difference in litter size. Analysis of male fertility showed that, in the Wfs1KO group, eight males out of 13 had pups whereas in the control group all 13 males had at least one litter. Sperm motility was not affected in Wfs1KO mice, but Wfs1KO males had less proximal bent tails (14.4 +/- 1.2% vs. 21.5 +/- 1.3 p < 0.05) and less abnormal sperm heads (22.8 +/- 1.8 vs. 31.5 +/- 3.5, p < 0.05) than wt males. Testes histology revealed significantly reduced number of spermatogonia (23.9 +/- 4.9 vs. 38.1 +/- 2.8; p < 0.05) and Sertoli cells (6.4 +/- 0.5 vs. 9.2 +/- 1.0; p < 0.05) in Wfs1KO mice. Serum testosterone and FSH concentrations did not differ between the two groups.

**Conclusion:**

The impaired fertility of Wfs1KO male mice is most likely due to changes in sperm morphology and reduced number of spermatogenic cells. The exact mechanism through which the Wfs1 gene influences sperm morphology needs to be clarified in further studies.

## Background

Wolfram syndrome (WS), also known as DIDMOAD syndrome, was first described by Wolfram and Wagener in 1938. It is an autosomal recessive disorder usually diagnosed in childhood when non-autoimmune type I diabetes occurs with optic atrophy, cranial diabetes insipidus and sensorineural deafness [1,2]. Other abnormalities related to this syndrome are dilated renal outflow tracts, multiple neurological abnormalities and various neurological and psychiatric disorders [2-5]. Involvement of the hypothalamus, brain stem (central sleep apnoea), and cerebellum (ataxia) may develop in the third decade or later [1].

Wolfram syndrome is caused by mutation in the *Wfs1 *gene on chromosome 4p16 [6]. This gene is responsible for encoding wolframin, a glycoprotein of the endoplasmic reticulum, although the function of the wolframin protein is not fully understood [7-9]. There is growing evidence that *Wfs1 *plays an important role in the pathogenesis of endoplasmic reticulum (ER) stress and apoptosis [8-10]. Genetic association studies have also indicated the role of *Wfs1 *in the development of type 2 diabetes [11].

As yet there has been no data regarding the fertility of patients with WS. Previous studies have described anterior pituitary dysfunction [5] and, in male patients, the presence of primary gonadal atrophy and hypergonadotropic hypogonadism [2-5]. As far as we know, the role of the *Wfs1 *gene in fertility has not been studied.

Mice lacking the *Wfs1 *gene (Wfs1KO) were created at the Laboratory of Physiology, University of Tartu [12]. This animal model of WS is useful to study the various organ-systems of WS, including fertility.

The aim of our study was to determine whether the fertility of Wfs1KO male mice is reduced and if so, to explore possible reasons. Regarding the possible causes of impaired fertility, we have focused on sperm morphology.

## Methods

### Animals

In accordance with the European Communities Directive (86/609/EEC), the Estonian National Board of Animal Experiments granted permission (No. 86, 28.08. 2007) for the animal experiments described in this study. Mice were housed under standard laboratory conditions on a 12-hour light/dark cycle (lights on at 07:00 hours) with free access to food and water.

**Figure 1:**
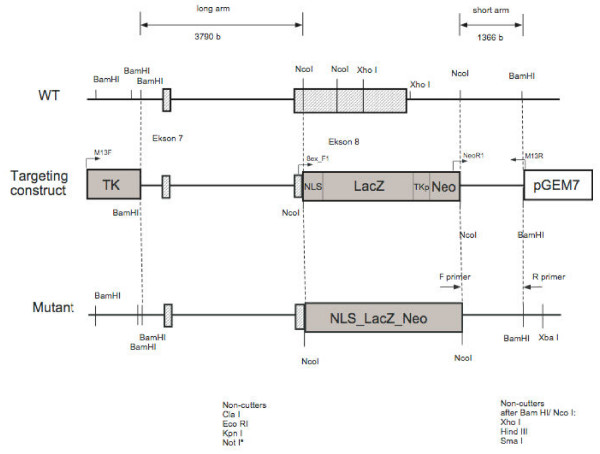
**The Wfs1 targeting vector was designed to replace exon 8 in the wfs 1 gene with the NLS-LacZ-Neo expression cassette**.

**Figure 2:**
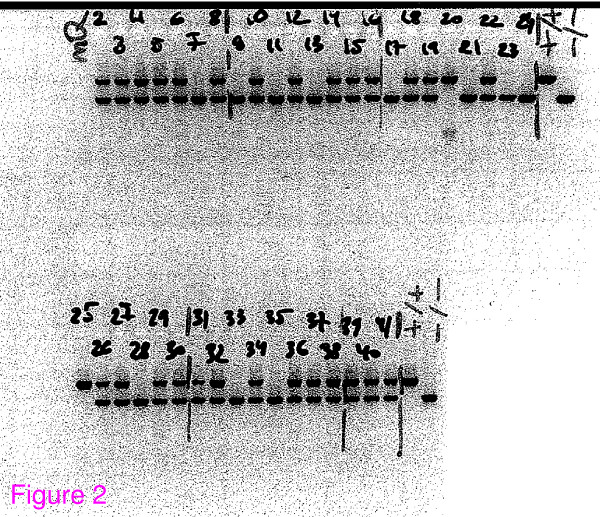
**Gel electrophoresis of PCR product to genotype wfs1 targeting products**. Mice were genotyped by multiplex PCR for both alleles using primers Wfs1KO_wf2 5' TTGGCTTGTATTTGTCGGCC 3', NeoR1 5' GACCGCTATCAGGACATAGCG 3' and WfsKO_uniR2 5' CCCATCCTGCTCTCTGAACC 3'. The upper band is for the wild-type allele; the lower band is for the mutant allele. The presence of two bands indicates a heterozygous mutant mouse.

**Figure 3:**
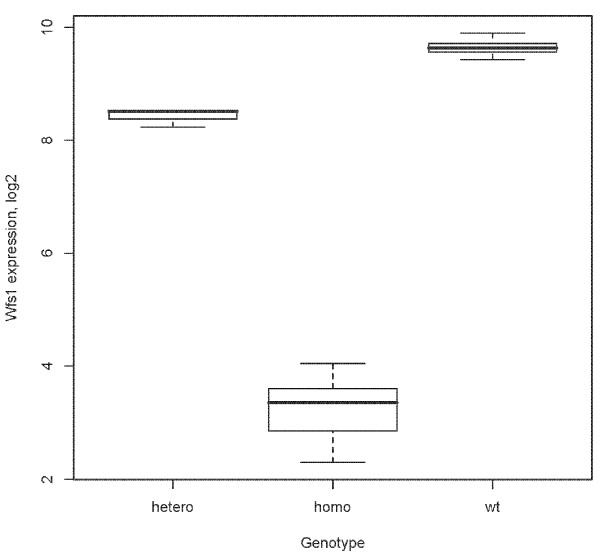
***Wfs1 *mRNA expression is reduced in mutant mice. Genechip data (log2 transformed) from mice with three different genotypes illustrate the almost complete lack of Wfs1 transcripts in homozygous mutant mice**. In heterozygous mice the *Wfs1 *mRNA level is half of that in wild-type mice.

*Wfs1 *deficient (Wfs1KO) mice were generated by targeting construct to replace most of the coding region of the *wfs*1 gene (Figure 1). Briefly, the 8.8 kb *BamHI *restriction fragment from the PAC clone 391-J24 (RPCI21 library, MRC UK HGMP Resource Centre, UK) was subcloned into a pGem11 cloning plasmid (Promega, Madison, WI). We replaced the 3.7-kb *Nco*I fragment with an in-frame NLSLacZNeo cassette. This resulted in the deletion of amino acids 360–890 in the *Wfs1 *protein and a fusion between the *Wfs1 *1–360 fragment and LacZ. This construct was inserted into W4/129S6 embryonic stem (ES) cells (Taconic, Hudson, NY) at the Biocenter of the University of Oulu . Colonies resistant to G418 and gancyclovir were screened for homologous recombination by polymerase chain reaction (PCR) by using the recombination-specific primers NeoR1 5'GACCGCTATCAGGACA TAGCG3' and Wfs1_WTR1 5'AGGACTCAGGTTCTGCCTCA3' (Figure 2). We sequenced the PCR product to verify that homologous recombination took place, and injected ES clone 8A2 into C57BL/6 blastocysts. The invalidation of Wfs1 gene was verified by mRNA expression analysis and we confirmed the lack of Wfs1 transcript in homozygous Wfs1 mutant mice [13] (Figure 3).

According to the Mouse Genome Database , the official designation of this strain is Wfs1^tm1Koks^. In order to avoid the "congenic footprint" effect [14] we bred animals into two different backgrounds and only used mice with the isogenic 129S6 background.

### Fertility study

We used 13 Wfs1KO, 13 wild type (wt) male mice and 156 wt female mice. All mice were 8–12 weeks old. All male mice, both wt and Wfs1KO, were housed in one cage with two wt female mice each. Every morning the females were checked for the presence of vaginal plugs, an indication that sexual activity had taken place. If a vaginal plug was present, the female was taken away from the cage and placed in another one. If there was no vaginal plug after three days, the mice were separated and two other females were introduced to the males. This was done three times. Thus, at the end of the period, six females had been introduced to each male mouse (6 × 13 × 2). Each week, every female was weighed and if they had gained weight they were transferred to a single cage, where they delivered their pups. The fertility rate and the size of the litter were counted for each male.

### Sperm morphology and motility study

Sperm were obtained from the cauda epididymides of mature male mice (11 Wfs1KO and 12 wt) previously killed by cervical dislocation. Two hundred spermatozoa per male were analysed, totalling 2200 spermatozoa in the Wfs1KO group and 2400 in the control group. Sperm motility was observed and recorded by CASA (Sperm Vision™, Minitübe, Germany). The percentage of motile spermatozoa and straight line motile spermatozoa was calculated. Sperm morphology was studied on wet preparations made from formol-saline fixed samples, under phase-contrast microscope at 1000× magnification. The sperm head morphology, sperm tail morphology and the presence of cytoplasmic droplets were studied using the methodology described by Kawai *et al*. (2006). Using sperm head morphology, the percentage of spermatozoa with abnormal sperm heads, including triangular, collapsed and hammer heads or with a hairpin at the neck, was calculated. Using sperm tail morphology, sperm tails were classified into three categories: straight tail, proximal bent tail and distal bent tail including angled and hairpin forms. The percentage of every form was calculated. The percentage of spermatozoa with none, light-type or heavy-type cytoplasmic droplets (CD) was also calculated.

### Testes histology

The structure of both testes of three wild-type mice and three Wfs1KO mice were analyzed (totalling 12 testes). Samples were fixed in 10% buffered formalin and embedded in paraffin according to routine methods. Specimens were cut at 4 μm thickness and stained with hematoxylin and eosin for examination by light microscopy. Specific cell counts were performed in each testicle in the seminiferous epithelium of five round-shaped seminiferous tubules, i.e. cells were counted in 10 tubules per mouse. Two independent, blind observers performed the cell counts.

### Measurement of hormonal levels

Blood samples were taken from mice after they were killed by cervical dislocation. Samples were centrifuged for 15 minutes at 1000 × g, 4 degrees. Serum was removed and samples analysed by ELISA kits (USCNLIFE, China) for testosterone and follicle-stimulating hormone (FSH). The optical density of the wells was determined with the ELISA reader SUNRISE (Tecan, Switzerland).

### Statistical analysis

All data was analysed using the statistical software package, SAS version 9.1 (SAS Institute Inc, Cary, North Carolina, USA). The Chi-square test or Fisher's Exact Test (when expected values were <5%) was used to compare the fertility rates, and the Student's t-test was used to compare the sperm morphology, litter size, occurrence of the vaginal plugs and concentrations of the hormones between the groups. Mean ± SEM are shown. P values < 0.05 were considered statistically significant.

## Results

### The fertility study

**Figure 4:**
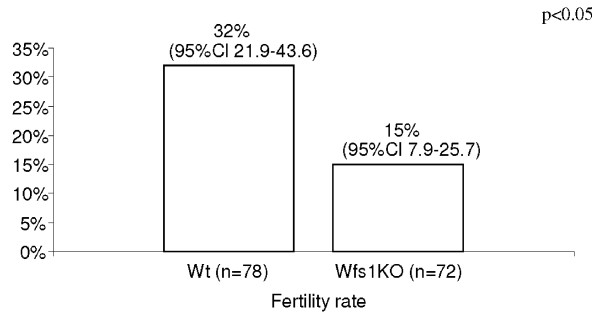
**The fertility rate in percentages, with 95% CI in brackets, in 72 female wt mice mated with Wfs1KO (n = 12) male and in 78 female wt mice mated with wt male mice (n = 13)**.

The pregnancy rate in female mice mated with Wfs1KO males tended to be lower than in those mated with wt males: 15/78 (19%; 95%CI 11.5 – 30.0) vs. 25/78 (32%; 95%CI 22.9 – 43.6), p = 0.1. We noticed that in the Wfs1KO group there was one male who had pups with four out of the six females. This was more than 3 SD above the group's mean. When this male and his six females were excluded from the analysis, the pregnancy rate in females mated with Wfs1KO males was significantly lower than the pregnancy rate in the control group: 11/72 (15%; 95%CI 7.9 – 25.7) vs. 25/78 (32%; 95%CI 21.9 – 43.6). P < 0.05; (Figure 4).

In analysing the fertility of Wfs1KO males, we found that five out of 13 of them did not have any litter, whereas all 13 of the control males had at least one litter (p < 0.05). There was no significant difference in the litter size: 5.7 ± 0.5 pups in the Wfs1KO group vs. 6.6 ± 0.5 pups in the wt group. When analysing the occurrence of vaginal plugs we found that out of the six female mice who were mated with one male mouse, vaginal plugs occurred in 1.1 ± 0.2 females in the Wfs1KO group, compared to their occurrence in 2.5 ± 0.4 females out of six in the wt group (p < 0.05).

### Sperm motility and morphology study

Sperm motility was not affected in Wfs1KO mice. Surprisingly, the mean percentage of motile sperm was even higher in the Wfs1KO mice than in the wt mice, whereas no statistical differences were observed in the percentage of straight motility (Table 1).

**Table 1:** Sperm morphology in male mice according to Kawai et al (2006).

***Characteristic***	***Wfs1KO (n = 11)***	***wt (n = 12)***	***p-value***
Motility	78.0 ± 2.8%	70.0 ± 3.4%	0.04
Straight motility	66.0 ± 3.5%	58.0 ± 4.4%	0.08
Sperm without CD	57.2 ± 4.9%	68.7 ± 5.4%	0.07
Light CD	30.5 ± 3.3%	22.5 ± 3.8%	0.07
Heavy CD	12.3 ± 1.8%	8.8 ± 1.8%	0.09
Straight tail	53.1 ± 1.5%	50.3 ± 1.7%	0.1
Proximal bent tail	14.4 ± 1.2%	21.5 ± 1.3%	0.0003
Distal bent tail	32.5 ± 2.3%	28.2 ± 1.7%	0.07
Hairpin at the neck	9.7 ± 0.7%	9.7 ± 0.8%	0.5
Abnormal head	22.8 ± 1.8%	31.5 ± 3.5%	0.02

Percentage of spermatozoa (out of 200) having the specific characteristic (mean ± SEM). (CD – cytoplasmic droplets).

**Figure 5:**
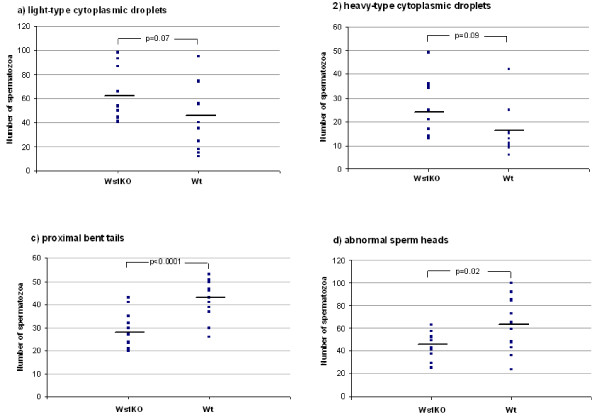
**Four most important abnormalities in sperm morphology: a) light-type cytoplasmic droplets; b) heavy-type cytoplasmic droplets; c) proximal bent tails and d) abnormal sperm heads**. The number of spermatozoa (out of 200) of each mouse having the specific characteristic is shown in dots. The mean number of the group is shown with a bold line.

The sperm morphology study showed that Wfs1KO males had fewer proximal bent tails than wt males, but had fewer abnormal sperm heads than wt males (Table 1, Figure 5). The sperm of Wfs1KO mice also tended to have more cytoplasmic droplets, both light-type and heavy-type, but the difference was not statistically significant (Table 1, Figure 5).

### Testes histology

**Figure 6:**
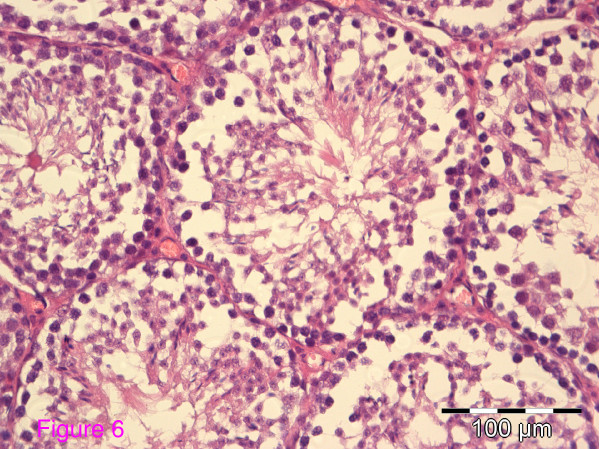
**Normal seminiferous epithelium of the seminiferous tubules in a wild-type mouse**.

**Figure 7:**
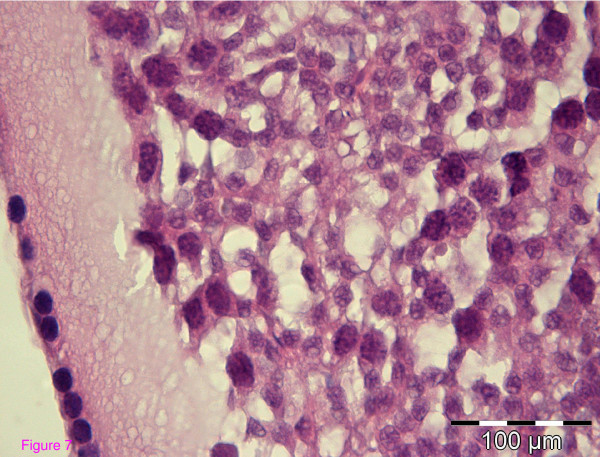
**Altered structure of the seminiferous epithelium in a Wfs1KO mouse**.

The organized architecture of the seminiferous epithelium of the seminiferous tubules seen in wt mice was lost in Wfs1KO mice (Figure 6). Contrary to the wild-type mice, the lumina of the seminiferous tubules in Wfs1-deficient mice have a typically irregular contour or the lumen may even be obliterated. Accumulation of eosinofilic luminal content is seen. Furthermore, several segments of these tubules have no spermatogenic cells at all (Figure 7). The seminiferous epithelium of Wfs1-deficient mice has reduced numbers of Spermatogonia and Sertoli cells, resulting in reduced sperm production (Table 2). There were no significant differences between the number and structure of Leydig cells between wt and Wfs1KO mice.

**Table 2:** The number of cells (mean and ± SEM) in the seminiferous epithelium of seminiferous tubules in wt and Wfs1KO mice.

***Group***	***SG***	***PSC***	***SSC***	***Spermatids***	***Sperms***	***Sertoli cells***
Control (n = 3)	38.1 ± 2.8	54.3 ± 0.8	2.1 ± 0.7	134.1 ± 6.1	35.4 ± 5.6	9.2 ± 1.0
Wfs1 (n = 3)	23.9 ± 4.9*	47.2 ± 7.0	0.0 ± 0.0	110.0 ± 15.2	13.3 ± 4.0*	6.4 ± 0.5*

SG – spermatogonia, PSC – primary spermatocytes, SSC – secondary spermatocytes.

*p < 0.05

### Serum hormone concentration

Serum testosterone and FSH concentrations in Wfs1KO males (12.0 ± 0.5 nmol/l and 5.6 ± 0.1 mlU/ml respectively) did not different significantly from those in wt males (11.5 ± 0.7 nmol/l and 5.8 ± 0.1 mlU/ml respectively).

## Discussion

We have shown for the first time that male Wfs1KO mice have reduced fertility compared to wt male mice. It is known that primary hypogonadism may occur in male patients with WS [2-5], but there has been no data about the fertility of patients with WS. One reason for this may be the few patients available with whom to carry out such a study, or the fact that other clinical symptoms, such as diabetes mellitus and vision or hearing impairments, dominate and precede the fertility problems. Consequently, we investigated whether Wfs1KO male mice have reduced fertility and, if so, their possible causes.

The pregnancy rate of wt female mice was lower when mated with Wfs1KO male mice, than when mated with wt males. All wt males used in our study gave at least one litter with one female out of six, whereas only eight Wfs1KO males out of 13 brought litters, indicating impaired fertility in male Wfs1KO mice. In order to examine the possible mechanisms causing reduced fertility, we examined the sperm of the same males used in the fertility study. Having studied the motility and morphology of the sperm in the Wfs1KO and wt groups, it was shown that the motility of spermatozoa in Wfs1KO mice was not impaired and was even slightly better than in the wt animals. However, many changes in sperm morphology were found. The most statistically significant difference was that the sperm of Wfs1KO mice contained fewer spermatozoa with proximal bent tails than the sperm of wt mice. The in vitro fertilization rate in mice has been shown to be positively correlated to proximal bent tails, but negatively to heavy-type CD and distal bent tail. These last two characteristics were higher in Wfs1KO mice compared to wt mice, but the difference did not reach statistical significance (p < 0.09 and 0.07 respectively). We did find that Wfs1KO males had fewer abnormal sperm heads. However, the impact of an abnormal sperm head on fertility has been found to be much smaller than the impact of different bent tails or cytoplasmic droplets as seen in the study by Kawai et al, in which a high percentage of abnormal sperm heads was related to a relatively good in vitro fertilization rate in mice [15].

Testicular histology showed a normal pattern of seminiferous epithelium in the tubules of wt mice, which was lost in Wfs1KO mice. Several segments of seminiferous tubules in Wfs1KO mice had no spermatogenic cells at all. The number of spermatogonia and Sertoli cells essential for effective spermatogenesis was decreased, leading to reduced sperm production. Thus, both sperm morphology and quantity is affected in Wfs1KO mice. However, the number and structure of Leydig cells responsible for testosterone synthesis did not different between the Wfs1KO and wt mice. This was also confirmed by normal testosterone levels in the two groups.

The mechanism by which wolframin deficiency may cause impaired fertility and changes in sperm morphology and quantity is not clear. One possible explanation may be through increased endoplasmic reticulum (ER) stress, which has been shown to cause progressive cell loss in pancreatic β-cells in Wfs1 deficient mice [10,16,17]. ER stress enhances *Wfs1 *gene expression and *Wfs1 *deficient mice are more susceptible to ER stress-induced apoptosis than wild type mice [17]. *Wfs1 *is also expressed in the testes of mice [18], so it is possible that increased ER stress in the testes of Wfs1KO mice may cause changes in spermatogenesis. Further studies, including electron microscopic studies of the endoplasmic reticulum of spermatozoa and the epithelium of seminiferous tubules in Wfs1KO mice, are necessary to confirm or reject our hypothesis.

Another possible reason for impaired fertility in Wfs1KO mice may be due to changes in the hypothalamic-pituitary axis. It is known that patients with WS have disturbed anterior pituitary function [5], and our laboratory has shown changes in the gene expression levels of GH, POMC and NPY in different parts of the brain [13]. Therefore, we also measured serum FSH and testosterone levels in these mice. We chose these two hormones because they both play an important role in spermatogenesis. However, we did not find any differences in their levels. Unfortunately we did not have enough serum to measure LH, but it is unlikely that serum LH concentrations would have been different due to comparable serum testosterone concentrations as well as the number and structure of Leydig cells. This observation suggests that it is unlikely that the impaired fertility in these mice would be caused by altered gonadotrophin levels. As the number of Sertoli cells was significantly lower in the Wfs1KO group, inhibin B should be measured in further studies.

We cannot exclude the possibility that a part of the impaired fertility on Wfs1KO male mice may be explained by different sexual behaviour. It is known that patients with WS frequently have psychiatric problems [19] and therefore may have defects in sexual behaviour. The occurrence of vaginal plugs in female mice was significantly lower when mated with Wfs1KO males, in comparison to wt males. However, the occurrence of vaginal plugs is not a very reliable method, since the plug lasts only couple of hours and can easily be missed. Specially designed studies with video recording are necessary to clarify whether sexual behaviour in Ws1KO mice is different from wild-type mice.

## Conclusion

In conclusion, Wfs1KO male mice have impaired fertility, most likely due to changes in sperm morphology and reduced number of spermatogenic cells. The exact mechanism how the *Wfs1 *gene and its product, wolframin, influence sperm morphology needs to be clarified in further studies.

## Competing interests

The authors declare that they have no competing interests.

## Authors' contributions

KN conceived the study along with SK and VT, participated in its design and coordination and drafted the manuscript. AnK was responsible for the sperm morphology and motility study, and manuscript preparation. AA and MA were responsible for the histological study of the testes. AiK carried out the fertility study. EV supported the conducting of the studies. SK and VT conceived the study along with KN, participated in its design and coordination and helped to draft the manuscript. All authors read and approved the final manuscript.
